# Draft Genome Sequence of Pseudomonas sp. Strain MWU13-3659, Isolated from Commercial Cranberry Bog Soil in Massachusetts, USA

**DOI:** 10.1128/mra.00880-22

**Published:** 2022-10-17

**Authors:** Amritpal Kooner, Scott Soby

**Affiliations:** a Chicago College of Osteopathic Medicine, Midwestern University, Downers Grove, Illinois, USA; b Biomedical Sciences, College of Graduate Studies, Midwestern University, Glendale, Arizona, USA; c College of Veterinary Medicine, Midwestern University, Glendale, Arizona, USA; University of Arizona

## Abstract

Pseudomonas sp. strain MWU13-3659 was isolated from cultivated cranberry bog soil in Massachusetts, USA. Its closest known relative is Pseudomonas entomophila (digital DNA-DNA hybridization [d4 formula] value of 57.2% and average nucleotide identity based on BLAST value of 93.90), and its genome contains putative gene clusters for the production of polyketides, siderophores, and cyclic lipopeptides that have insecticidal activity in other proteobacteria.

## ANNOUNCEMENT

Pseudomonas spp. constitute a major proportion of bacterial isolates in a multiyear culture-dependent survey of wild and cultivated cranberry bogs (1–8). Wetland microecosystems in general, and the microbiomes of cranberry bogs in particular, are largely unexplored, and little is understood about the role of pseudomonads in those environments. MWU13-3659 was isolated from cultivated cranberry bog soil at the University of Massachusetts State Bog (41.766767N, 70.66842W) in early July 2013. A ~1 g sample from a soil core (5 cm by 5 cm) was vortex-mixed in 10 mL sterile distilled water, and the rinsate was plated on King’s medium B (KMB) agar containing 50 μg mL^−1^ each of ampicillin and cycloheximide. Individual fluorescent colonies were picked onto fresh KMB agar, single colony purified three times, and stored at −80°C in 34% glycerol. MWU13-3659 was recovered from storage by plating on KMB agar, and then a population was inoculated into overnight KMB broth cultures for genomic DNA isolation with a DNeasy blood and tissue kit (Qiagen, USA). Kits used in this work were used as instructed by their manufacturers. Illumina-compatible genomic DNA libraries were generated using a HyperPlus library preparation kit (Kapa Biosystems product number KK8514; Roche, USA). DNA was enzymatically sheared to ~500 bp, end repaired, A-tailed, ligated to Illumina-compatible adapters (product number 00989130v2; Integrated DNA Technologies, Coralville, IA), cleaned using KAPA pure beads (Kapa Biosystems product number KK8002), and amplified with KAPA HiFi enzyme (Kapa Biosystems product number KK2502). Library fragments were sized on an Agilent TapeStation system, quantified by quantitative PCR (KAPA library quantification kit [Kapa Biosystems product number KK4835]) on a QuantStudio 5 system (Thermo Fisher Scientific, USA), multiplex pooled, and sequenced in a 2 × 250 bp flow cell using the Illumina MiSeq platform. All software was used with default settings except as indicated. Raw reads were assembled with Unicycler v0.4.8 within the PATRIC (https://www.bv-brc.org/) Comprehensive Genome Analysis pipeline v3.6.12, with the trim setting set to true (9, 10). The Comprehensive Genome Analysis pipeline includes polishing by Pilon v1.23 (11), quality control and trimming by QUAST v5.0.2 (12) and Trim Galore v0.4.0 (13), and annotation by RASTtk v1.073 (14). MWU13-3659 was placed in the genus Pseudomonas by Type Strain Genome Server (TYGS) analysis (15), but the closest relative was Pseudomonas entomophila L48^T^ (16), with a digital DNA-DNA hybridization (dDDH) (d4 formula) value of only 57.2% and an average nucleotide identity based on BLAST (ANIb) value of 93.90% determined by JSpeciesWS v3.9.5 (17), which are well below the accepted species-level cutoff values of 70% and 95 to 96%, respectively (18–21). The genome of MWU13-3659 was 6,284,185 bp assembled from 3,055,790 reads into 132 contigs, with an *N*_50_ value of 229,522 bp and a G+C content of 63.85% from a total read length of 726,238,536 bp, giving coverage of 115×. The genome contains putative genes for the synthesis of multiple polyketides and cyclic lipopolypeptides with the potential for biological activity against insects, including the siderophore pseudomonine, entolysin, rhizomides, and sessilin (22–25).

### Data availability.

This whole-genome sequence project has been deposited in DDBJ/EMBL/GenBank under BioProject accession number PRJNA691338, BioSample accession number SAMN27103138, and genome accession number JAMSHW000000000. The version described in this paper is JAMSHW000000000.1. The raw reads are available in the Sequence Read Archive (SRA) under accession number SRR18741572. RASTtk annotations are available under open license at Zenodo (https://zenodo.org/record/6458518#.YwQD90fMKUk).
